# Alteration of Dynein Function Affects α-Synuclein Degradation via the Autophagosome-Lysosome Pathway

**DOI:** 10.3390/ijms141224242

**Published:** 2013-12-13

**Authors:** Da Li, Ji-Jun Shi, Cheng-Jie Mao, Sha Liu, Jian-Da Wang, Jing Chen, Fen Wang, Ya-Ping Yang, Wei-Dong Hu, Li-Fang Hu, Chun-Feng Liu

**Affiliations:** 1 Suzhou Key Laboratory of Translational Research of Neuro-Psycho-Diseases and Department of Neurology, Second Affiliated Hospital of Soochow University, 1055 Sanxiang Road, Suzhou 215004, China; E-Mails: huiyue999@gmail.com (D.L.); shijijun2008@126.com (J.-J.S.); drchengjiemao@163.com (C.-J.M.); lululiusha@163.com (S.L.); wangjianda611@gmail.com (J.-D.W.); jing_ch.china@hotmail.com (J.C.); yapingyang2005@163.com (Y.-P.Y.); weidonghu@aliyun.com (W.-D.H.); 2 Institute of Neuroscience, Soochow University, 199 Ren-Ai Road, Suzhou Industrial Park, Suzhou 215123, China; E-Mail: wangfen_1982@126.com

**Keywords:** dynein, α-synuclein, Parkinson’s disease, autophagy, autolysosome

## Abstract

Growing evidence suggests that dynein dysfunction may be implicated in the pathogenesis of neurodegeneration. It plays a central role in aggresome formation, the delivery of autophagosome to lysosome for fusion and degradation, which is a pro-survival mechanism essential for the bulk degradation of misfolded proteins and damaged organells. Previous studies reported that dynein dysfuntion was associated with aberrant aggregation of α-synuclein, which is a major component of inclusion bodies in Parkinson’s disease (PD). However, it remains unclear what roles dynein plays in α-synuclein degradation. Our study demonstrated a decrease of dynein expression in neurotoxin-induced PD models *in vitro* and *in vivo*, accompanied by an increase of α-synuclein protein level. Dynein down-regulation induced by siRNA resulted in a prolonged half-life of α-synuclein and its over-accumulation in A53T overexpressing PC12 cells. Dynein knockdown also prompted the increase of microtubule-associated protein 1 light chain 3 (LC3-II) and sequestosome 1 (SQSTM1, p62) expression, and the accumulation of autophagic vacuoles. Moreover, dynein suppression impaired the autophagosome fusion with lysosome. In summary, our findings indicate that dynein is critical for the clearance of aberrant α-synuclein via autophagosome-lysosome pathway.

## 1. Introduction

Alterations in axonal transport represent early but critical pathogenic events in neurodegenerative disease like Parkinson’s disease (PD) [1]. However, the molecular mechanisms underlying these defects remain elusive. Cytoplasmic dynein (later referred to as dynein) is one of the transport proteins that power ciliary beating, construct the mitotic spindle, and transport intracellular cargos such as injured mitochondria and misfolded proteins from the periphery to the cell center in a process called “retrograde transport” [2]. Given the essential role of dynein in many basic cell processes, its abnormality may have potential consequences like decreased transport of organelles and retrograde signaling back to the cell body [3]. Mutations that affect dynein motor machinery are sufficient to cause motor neuron diseases. A number of dynein functions have also been, directly or indirectly, linked to neurodegenerative diseases, including PD. *p150* is a gene which encodes a subunit of dynein complex. Mutation in *p150* is related to an atypical PD resistant to L-DOPA, called Perry syndrome [4]. Familial PD related gene *parkin* bound mitochondria to dynein through the histone deacetylases (HDACs) called HDAC6. This docking triggers their transport to the aggresome in dynein-dependent transportation [5].

PD is pathologically featured by the formation of cytoplasmic inclusion bodies in the remaining dopaminergic neurons in midbrain and other affected regions. α-synuclein is the main component of inclusion bodies. Autophagy-lysosome pathway (ALP) is critical for clearing mutant α-synuclein. ALP impairment contributes to PD pathogenesis [6–8]. ALP is a multistep process, consisting of double-membraned autophagosome formation and maturation, autophagosome fusion with lysosome, and degradation or recycling of cytoplasmic constituents [9,10]. Autophagosomes move in a microtubule- and dynein-dynactin motor complex-dependent manner. Dynein prompts autophagosomes moving towards the cell soma, where most lysosomes are located, and thus mediates efficient autophagosomes encountering with lysosomes [11]. As autophagosomes move distally toward proximally, they undergo maturation and become increasingly acidified. In this maturation and fusion progress, dynein and kinesin are also involved [12]. More importantly, Chu *et al.* [13] demonstrated the decreases of dynein light chain Tctex type 3 DYNLT3 in the late stage but not in the early stage of PD. Moreover, the reductions of DYNLT3 levels were selectively associated with accumulated α-synuclein inclusions. These changes were recapitulated in a rat model of familial PD based on over-expression of human mutant α-synuclein (A30P) [13]. These lines of evidence strongly suggest that defects in dynein complex may lead to an accumulation of α-synuclein within dopaminergic neurons. However, the molecular mechanisms are less well understood.

Our previous study showed that, after 1-methyl-4-phenylpyridinium (MPP^+^) treatment, dynein expression decreased, mainly aggregated at the periphery of cytoplasm, and lost its colocalization with α-synuclein and the lysosome marker lamp1 [14]. It is therefore tempting to speculate that the downregulation of dynein function may result in ALP disruption and eventually lead to excessive accumulation of α-synuclein in PD. To this end, we examined the expression of dynein in different toxin-induced PD models and explored the possible role and the underlying mechanisms of dynein in α-synuclein clearance by genetic manipulation of this motor protein.

## 2. Results and Discussion

### 2.1. Dynein Expression Is Decreased in Neurotoxins-Treated PC12 Cells

**Figure 1.**
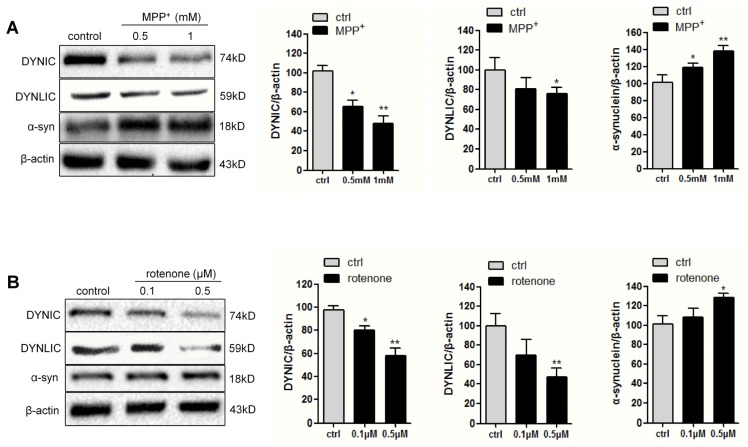
Neurotoxin-induced a decrease of dynein chains but increase of α-synuclein in PC12 cells. Cells were treated with 0.5 or 1 mM 1-methyl-4-phenylpyridinium (MPP^+^) (**A**); 0.1 or 0.5 μM rotenone (**B**) or vehicle for 24 h. 20 μg proteins were analyzed for dynein intermediate chain (DYNIC), dynein light intermediate chain (DYNLIC) and α-synuclein immunoreactivity with β-actin as loading controls. Data are expressed as mean ± SEM, *n* = 3. *p < 0.05; **p < 0.01 *versus* controls.

To examine the possible role of dynein in PD pathogenesis, we first evaluated the protein level of both dynein intermediate chain (DYNIC) and light intermediate chain (DYNLIC) in different toxin-induced PD models in PC12 cells after exposure to MPP^+^ (0.5 and 1 mM) or rotenone (0.1 and 0.5 μM) for 24 h. As shown in Figure 1, the levels of both chains, namely, DYNIC and DYNLIC, were decreased in the neurotoxins-treated cells. Quantitative analysis revealed that anti-DYNIC immunoreactivity was reduced about 50% and 40%, while anti-DYNLIC immunoreactivity about 20% and 40%, respectively, when 1 mM MPP^+^ and 0.5 μM rotenone were added. Not surprisingly, MPP^+^ and rotenone treatment also led to the increase of α-synuclein protein level.

### 2.2. The α-Synuclein Clearance Is Impaired in Dynein-Silenced PC12 Cells

**Figure 2.**
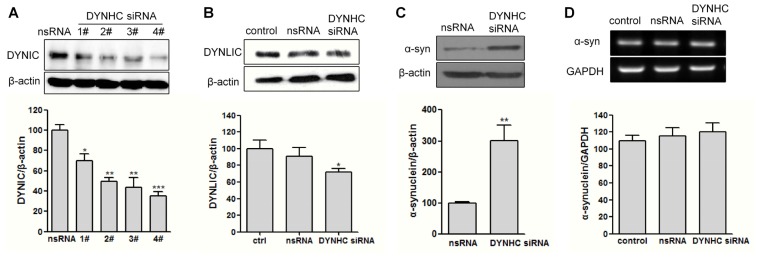

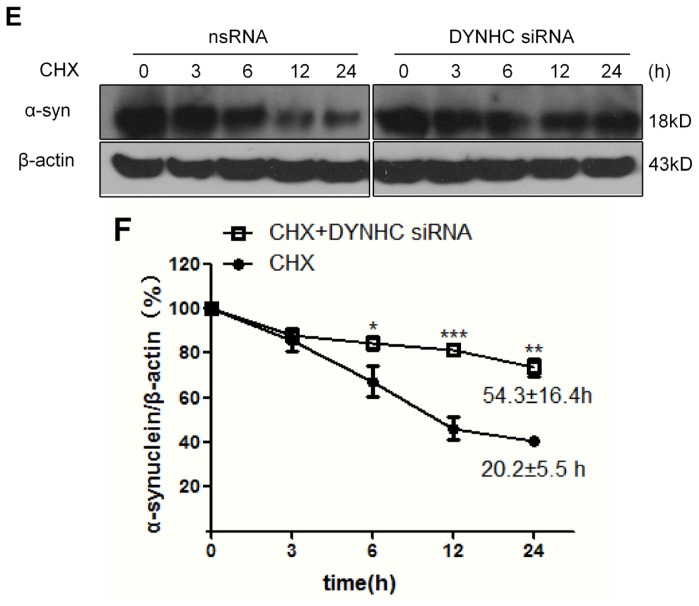
The clearance of mutant α-synuclein A53T is impaired in dynein knocked down cells. (**A**,**B**) A53T stably expressing PC12 cells were transfected with siRNAs against DYNHC or scrambled RNA (nsRNA). The knockdown efficiency was determined by western blotting at 48 h after transfection; (**C**,**D**) The α-synuclein (α-syn) protein expression and its mRNA level were determined at 48 h after transfection; and (**E**,**F**) 1 μg/mL CHX was added into the culture medium for up to 24 h to inhibit protein biosynthesis after siRNA transfection. Cells were harvested and lysed at 0, 3, 6, 12, and 24 h after CHX treatment. The protein degradation rate is expressed as half-life (*t*_1/2_), the time for degradation of 50% of α-synuclein protein. Data represent mean ± SEM, *n* = 3, *p < 0.05; **p < 0.01; ***p < 0.001 *versus* the indicated control group.

To determine whether the α-synuclein clearance is affected by dynein down-regulation, we continued to evaluate the α-synuclein expression in dynein-suppressed cells by RNA interfering technique. Dynein is a large, multimeric protein complex composed of two heavy chains, two intermediate chains, four light intermediate chains and light chains. The dynein heavy chain (DYNHC) contains the ATPase activity and microtubule binding domains, thus we chose DYNHC as knockdown target to block dynein complex function. The RNA interfering efficiency was determined at 48 h after siRNA transfection. Unfortunately, we failed to detect DYNHC by western blot, probably due to the high molecular weight of this chain, which is around 530 kDa. Nevertheless, all the four tested siRNAs were able to reduce both the DYNIC and DYNLIC levels, as compared to the scrambled RNA (nsRNA) transfected group (Figure 2A,B). This indicates the dynein complex function may be suppressed in the DYNHC-silenced cells. This is consistent with Kimura’s study [15]. Moreover, the knocking down efficiency of No.4 siRNA reached more than 60%. Therefore, this sequence of dynein siRNA was used in the following study.

Moreover, DYNHC knockdown was found to significantly enhance the protein expression rather than mRNA level of α-synuclein in A53T α-synuclein stably transfected PC12 cells (Figure 2C,D). To further determine whether the increased α-synuclein level was caused by degradation impairment, cycloheximide (CHX), a protein translation inhibitor, was used. As shown in Figure 2E, CHX, which alone decreased the α-synuclein protein level in nsRNA-transfected cells, prolonged the α-synuclein half-life (*t*_1/2_) with DYNHC knockdown. Specifically, its half-life in nsRNA-transfected cells was about 20.2 ± 5.5 h, but it rose to 54.3 ± 16.4 h in dynein knocked down cells (Figure 2F). This indicates the α-synuclein clearance may be impaired when dynein function is suppressed.

### 2.3. Autophagy Is Activated in Rotenone-Intoxicated PC12 Cells and Rat Striatum

**Figure 3.**
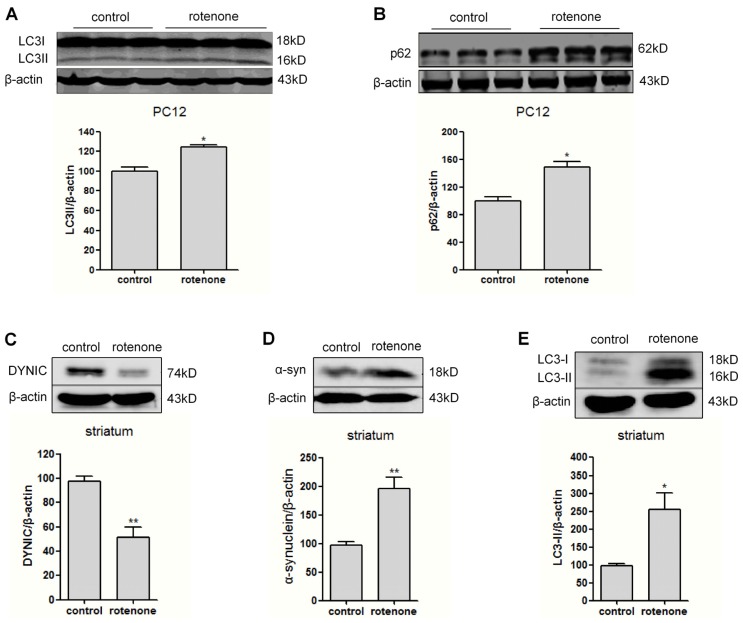
Upregulation of autophagic markers LC3-II and p62, associated with DYNIC decreases in rotenone-intoxicated PC12 cells and rat striatum. (**A**,**B**) LC3II and p62 levels were determined by western blot in PC12 cells exposed to 0.5 μM rotenone or vehicle for 24 h; and (**C**–**E**) Striatum was isolated from animals sacrificed at 30 days after subcutaneous injection of either vehicle or rotenone at a low dose of 1.0 mg kg^−1^·d^−1^. Protein levels of DYNIC, α-synuclein and LC3 from three independent rat striatum were examined by western blot. Results were expressed as mean ± SEM, *n* = 3. * *p* < 0.05; ** *p* < 0.01 *versus* controls.

ALP is a critical route for α-synuclein degradation [16]. We continued to examine the levels of autophagic markers LC3II and p62. In 0.5 μM rotenone-treated PC12 cells, an elevation of LC3-II and p62 level was observed (Figure 3A,B), indicating that autophagy was activated but with autophagic flux disruption. To verify the *in vitro* finding, the levels of dynein and α-synuclein were examined by western blotting in the striatum of rats following subcutaneous administration of rotenone. Our previous work demonstrated that after injection with rotenone at 1.0 mg kg^−1^·d^−1^ for 30 days, the rats exhibited movement dysfunction. More important, the dopaminergic neuron loss in substantia nigra compacta reached about 25%, and α-synuclein aggregates were found in the remaining dopaminergic neurons [17]. Consistently, we found in this study that α-synuclein level was obviously enhanced in the rat striatum after rotenone intoxication for 30 days (Figure 3D). Moreover, we observed a marked decrease of DYNIC in the rotenone-intoxicated rat striatum (Figure 3C). In addition, the LC3-II level also increased after rotenone injection (Figure 3E).

### 2.4. Autophagic Flux Is Disrupted in Dynein Silencing PC12 Cells

**Figure 4.**
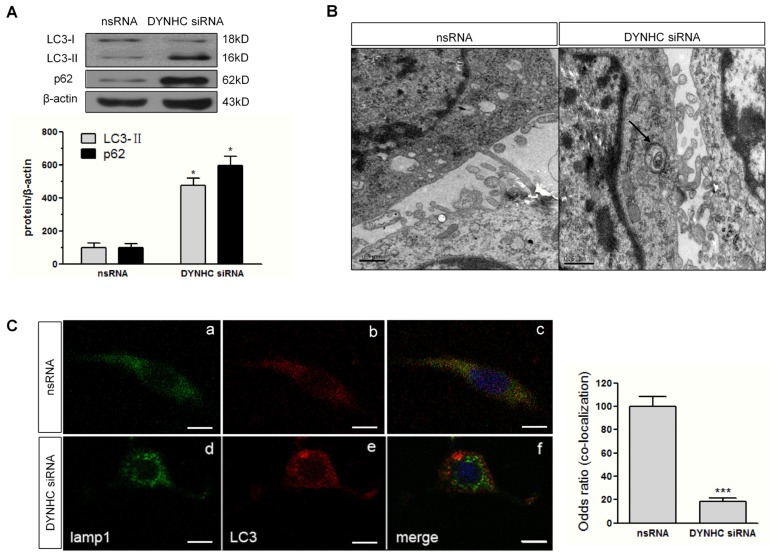
Autophagic flux is impaired in dynein-silencing PC12 cells. (**A**) LC3-II and p62 levels were determined by western blotting at 48 h following dynein knockdown in PC12 cells. Graphs represent the quantification of protein expression relative to actin from three independent blots; (**B**) Electron microscopy examination of autophagosome formation at 48 h after dynein knockdown. Arrows indicate typical autophagosomes in PC12 cells with dynein knockdown. Photos were taken at 20,000× magnification. Scale bar: 0.5 μm; (**C**) Cells were stained for LC3 (red) and lamp-1 (green) in control (**a**,**b**) and dynein-RNAi group (**d**,**e**). **c** and **f** represents the co-localization of LC3 with lamp-1 in control (**c**) and dynein-RNAi group (**f**), respectively. Nuclei were stained with hoescht 33258 (blue). Photos were taken at 630× magnification. Scale bar: 10 μm. The number of autophagosome-lysosome fusion corpuscles (odds ratio) are shown in the panel. *p < 0.05 *versus* its corresponding controls; ***p < 0.001 *versus* controls.

We also examined the autophagic activity after dynein knockdown. As shown in Figure 4A, the autophagy-related proteins, LC3-II and p62 was markedly elevated in response to dynein silencing. In agreement, transmission electron microscopy study showed that the number of autophagosomes, featured by double-membraned compartment, was also increased in dynein silencing cells (Figure 4B). Furthermore, the extent of the lysosomal marker lamp1 and LC3 co-localization was significantly reduced in dynein knockdown group compared to controls, implying that dynein knockdown may impair the autophagosome fusion with lysosome (Figure 4D,E).

### 2.5. Discussion

Decreases of dynein expression and mutations of this protein, correlate with dopaminergic neuron degeneration in PD [13,18,19]. Dynein dysfunction is also linked to the impairment of α-synuclein clearance, which may be responsible for the vulnerability of dopaminergic neurons [20], although the pathogenic form of this protein remains an open issue so far. However, it is yet to be established whether the alterations in dynein function result in α-synuclein aggregation, or vice versa. Our present study demonstrated the dynein decrease in MPP^+^- and rotenone-induced *in vitro* and *in vivo* PD models, and showed that altered dynein function contributed to abnormal α-synuclein accumulation. Specifically, dynein siRNA transfection resulted in a prolonged half-life of α-synuclein and its accumulation in A53T overexpressing PC12 cells, and prompted the elevation of LC3II and p62 expression, and the accumulation of autophagic vacuoles. Dynein repression also impaired the autophagosome fusion with lysosome. Our findings suggest that dynein may be implicated in autophagosome-lysosome fusion, which is essential for α-synuclein degradation via ALP.

The cause-effect relationship between aggregated α-synuclein and dynein expression or function is poorly understood. Previous study showed that MPP^+^ induced a decrease in kinesin-dependent anterograde rates while enhanced the dynein-based retrograde transport. However, this increased motility depends on ATP hydrolysis and the exogenous ATP [21]. Recently, Chu *et al.* demonstrated that kinesin levels were markedly reduced in remaining nigral neurons in sporadic PD and this reduction occurs at very early stage [13]. The presence of α-synuclein inclusions exhibited much more decline in kenasin levels. In contrast, the downregulation of dynein DYNLT3 subunit levels were only observed at late stage. These alterations of axonal transport proteins were reproduced in a rat genetic PD model based on over-expression of A30P α-synuclein [13] In this study, both MPP^+^ and rotenone treatment caused α-synuclein accumulation and dynein reduction (Figure 1). The RNAi experiments further demonstrated that dynein depletion induced a significant increase of α-synuclein and that this increase was mainly related to impaired clearance (Figure 2). This provides the evidence that dynein function is critical for α-synuclein degradation. The increased α-synuclein may further lead to a defective autophagic clearance of the unwanted protein, which was poorly transported to its normal synaptic location and poorly cleared by perikaryal proteins such as LAMP1, the lysosomal protease cathepsin D and the 20S proteasome [22]. It is likely that the aberrant up-regulation of α-synuclein may perturb the dynein-mediated axonal transport [23], thus forming a “vicious circle” that eventually results in α-synuclein accumulation.

Efficient degradation of macromolecules via ALP requires intact microtubule cytoskeleton, the motor machinery of dynein and functional lysosome [24]. Dynein mediates intracellular trafficking of macromolecules and organells [25]. Thus, we hypothesized that dysfunction of dynein-mediated transport may disrupt the trafficking of autophagosome and lysosome, resulting in the impaired autophagic clearance of α-synuclein. In this study, we observed a specific and stable increase of autophagy marker LC3-II after dynein-silencing, and an increase in p62 level. This indicates the deficiency of autophagy flux, which was confirmed by the accumulation of autophagic vacuoles in the cytoplasm periphery of PC12 cells by electron microscope (Figure 4). Therefore, the impaired fusion of autophagosome with lysosome caused by dynein knockdown could be a reason for α-synuclein accumulation. Therefore, these results confirmed the linkage between dynein dysfunction and α-synuclein accumulation, further supporting the critical role of dynein in removing aberrant α-synuclein via autophagy.

Impaired autophagy by MPP^+^ is sufficient to induce α-synuclein accumulation. One might expect to see gained function of dynein may rescue the disrupted ALP, thus prompt the α-synuclein degradation. However, overexpression experiments for dynein are notoriously difficult. There is no definitive evidence that overexpression of a certain chain of dynein is considered as gain of dynein function. It is very likely that enhanced dynein function may lead to promote the autophagic degradation of α-synuclein. However, the functional assay of dynein complex requires to be validated further.

Dynein is a large, multimeric protein complex composed of two heavy chains, two intermediate chains, four light intermediate chains and several light chains. As part of the complex, each chain has its specific functions. For example, DYNLC is important for cargo binding specificity [26]. DYNIC and DYNLIC are involved in its recruitment to late endosomes and lysosomes [27]. Moreover, dynein complex activity requires a number of other proteins such as dynactin, which interacts with dynein through DYNIC and p150^Glued^. As the DYNHC contain the ATPase activity and microtubule binding domains, here we chose DYNHC as a knockdown target to block dynein complex function. However, probably due to its high molecular weight (around 530 kDa), we failed to detect the DYNHC by western blotting. Thus, we turned to examine DYNIC and DYNLIC in our PD models and dynein silencing cells, and found that both DYNIC and DYNLIC were reduced in neurotoxin-treated PC12 cells and DYNHC-silenced cells. How does this occur remains to be elucidated. But, this is consistent with Kimura’s report [15]. They demonstrated that after DYNHC siRNA introduction, the DYNIC expression also significantly decreased. Nevertheless, the specific relevance of other dynein subunits in autophagic degradation of α-synuclein deserves further investigation as well.

In addition to α-synuclein, dynein may have effect on other aggregate-prone proteins such as β-amyloid precursor protein and huntingtin [15,28]. For example, snapin knockdown neurons exhibit aberrant accumulation of immature lysosomes, clustering and impaired retrograde transport of late endosomes along processes, while snapin overexpression enhances late endocytic transport and lysosomal function [29]. Acetylated microtubules are required for fusion of autophagosome with lysosome to form autolysosome [30]. In support of this, our previous study reported that HDAC6 participated in the degradation of MPP^+^-induced α-synuclein aggregates by regulating ALP [31].

## 3. Experimental Section

### 3.1. Reagents and Antibodies

MPP^+^ and rotenone were purchased from Sigma-Aldrich (St. Louis, MO, USA). Cycloheximide was obtained from Beyotime (Nantong, China) and lipofectinamine 2000 from Invitrogen (Carlsbad, CA, USA). The sources for primary antibodies are listed as follows: LC3b, α-synuclein and lamp1 (Abcam, Cambridge, UK); p62 (Enzo, New York, NY, USA); DYNIC (Dallas, Santa Cruz, CA, USA); DYNLIC (Epitomic, Burlingame, CA, USA). The small interfering RNA (siRNA) targeting rat DYNHC was 5′-CCAAAUACCUACAUUACUU-3′ (Catalog No. D-080024-04) and non-sense (scrambled) siRNA were purchased from Dharmacon (Lafayette, CO, USA).

### 3.2. Cell Culture

Rat pheochromocytoma cell line PC12 was purchased from the Institute of Cell Biology, Chinese Academy of Sciences (Shanghai, China). The PC12 cell line stably expressing human mutant (A53T) α-synuclein was built up and described in our previous studies [31]. G418 (Life Technologies, Carlsbad, CA, USA) at 200 nM was routinely added in A53T PC12 cell culture. PC12 cells were cultured in RPMI1640 medium. All medium was supplemented with 10% FBS and 1% of penicillin/streptomycin at 37 °C in a humid 5% CO_2_/95% air environment. Most of the analyses were performed at 24 h post-transfection or treatment unless otherwise noted.

For small interfering RNA (siRNA) experimentation, cells were cultured in serum-free medium for 4 h in the absence of antibiotics, followed by transfection with 4 μg of the indicated plasmid or siRNA using Lipofectamine 2000 (Life Technologies, Carlsbad, CA, USA).

### 3.3. Animals

All male Sprague-Dawley rats (SD, weighing 200–225 g, 3-month-old) from Shanghai Laboratory Animal Center, Chinese Academy of Sciences (Shanghai, China) were used in this study. Rats were housed under standard conditions (SPF-grade animal room, 12 h light-dark cycles, 24 ± 1 °C, and 70% ± 4% relative humidity, stocking density ≤ 5/box). Water and food pellets were given ad libitum. Animals were randomly divided into two groups: control group and rotenone-treated group (*n* = 10 in each group). Rotenone administration and animal care were carried out in accordance with the guidelines outlined in the “Guide for the Care and Use of Laboratory Animals” [32] of the U.S. Department of Health and Human Services. All procedures performed were also approved by the Institutional Animal Care and Use Committee of Soochow University (Suzhou, China).

Rotenone was dissolved in 33% dimethylsulfoxide (DMSO; Sigma-Aldrich, St. Louis, MO, USA) in saline, and was subcutaneously (s.c.) injected at 1.0 mg kg^−1^·d^−1^ at 24 h intervals for 30 days as previously reported [17]. Animals in control group received vehicle (2:1, DMSO/saline). Rats were anesthetized by injection with ketamine (80 mg/kg, i.p.) and sacrificed by decapitation. Brain striatum tissues were dissected on ice and samples were subjected to western blotting analysis to determine the levels of dynein, α-synuclein and LC3.

### 3.4. Western Blot Analysis

Cell lysates were prepared using lysis buffer (150 mM NaCl, 25 mM Tris, 5 mM EDTA, 1% Nonidet P-40, pH7.5) with protease inhibitor cocktail tablets (Roche Diagnostics, Penzberg, Germany). Protein samples were separated on 10% sodium dodecyl sulfate-polyacrylamide gels and transferred onto polyvinylidene fluoride membranes (Millipore, Bedford, MA, USA). Next, membranes were blocked with 5% (*w*/*v*) dry milk powder in 0.1% Tris buffered saline/Tween 20 (TBST) for 1 h and incubated with primary antibodies at optimized dilutions at 4 °C overnight. After that, membranes were briefly washed and incubated with secondary antibodies for another 1 h. Specific proteins were detected using a chemiluminescence kit (GE Healthcare, Buckinghamshire, UK). The densitometric analysis was performed using Image J software (National Institute of Health, Bethesda, MD, USA).

### 3.5. Cycloheximide(CHX)-Based Protein Chase Experiment

A53T PC12 cells were transfected with dynein siRNAs, followed by 1 μg/mL CHX treatment at 24 h later. Cells were harvested and lysed at 0, 3, 6, 12, and 24 h after CHX exposure. Protein concentrations were determined by Bradford assays, and protein expression was detected by western blot. The protein degradation rate is expressed as half-life (*t*_1/2_), the time for degradation of 50% of α-synuclein protein.

### 3.6. Immunocytochemistry

Cells were fixed with methanol, followed by blocking with PBS containing 10% bovine serum albumin (Sigma-Aldrich, St. Louis, MO, USA). Next, cells were incubated with primary antibodies overnight at 4 °C. Cells were then washed with 0.1% Tween-20 in PBS for three times and incubated with Alexa Fluor^®^488 or 555 secondary antibodies (Life Technologies, Carlsbad, CA, USA). Hoechst 33342 (1 μg/mL; Sigma-Aldrich, St. Louis, MO, USA) was applied to stain nucleus. Negative controls were performed in slides probed with secondary antibody only. Images were taken and processed on a confocal microscope system (LSM 700, Carl Zeiss Inc., Oberkochen, Germany). Acquisition software was (zen 2011, Expert mode Carl Zeiss Inc., Oberkochen, Germany).

### 3.7. Electron Microscopy Study

Cells were grown on glass coverslips and fixed in ice-cold 2.5% glutaraldehyde in 0.1 M PBS (pH 7.4) for 2 h. After post fixation in 1% osmium tetroxide (Sigma-Aldrich, St. Louis, MO, USA), cells were stained with 2% uranyl acetate (Polysciences Inc., Taipei, Taiwan), dehydrated in ethanol, and then flat-embedded in Epon (Shell Company, Deer Park, TX, USA). Thin sections were stained with uranyl acetate and lead citrate (Sigma-Aldrich, St. Louis, MO, USA) and subsequently examined under the transmission electron microscope (JEM 1230, JOEL, Tokyo, Japan).

### 3.8. Reverse Transcription PCR (RT-PCR)

Total RNA was extracted with Trizol (Invitrogen, Carlsbad, CA, USA) following the manufacturer’s instructions. Total RNA (1 g) of each sample was reversely transcribed into cDNA using cDNA synthesis kit (Fermentas, Vilnius, Lithuania). An equal volume of the resulting cDNA product was amplified using PCR Master Mix kit (Fermentas, Vilnius, Lithuania) with primers (Genscript, Nanjing, China) as follows: α-SYN (rat), 5′-CCT CAG CCC AGA GCC TTT C-3′ (forward), 5′-CCT CTG CCA CAC CCT GCT T-3′ (reverse); GAPDH (rat), 5′-GTT TCT TAC TCC TTG GAG GCC AT-3′ (forward), 5′-TGA TGA CAT CAA GAA GTG GTG AA-3′ (reverse). PCR products were separated in 2% agarose gels. The optical band densities were analyzed with Image J software (National Institute of Health, Bethesda, MD, USA).

### 3.9. Data Analysis

All Data are expressed as mean ± SEM. Statistical analysis was performed by SPSS Version 6.1 software (SPSS, Chicago, IL, USA) using Student’s *t*-Test or one-way analysis of variance followed by Tukey *post hoc* analysis where applicable. Differences were considered significant when *p* value < 0.05.

## 4. Conclusions

Our findings provide the evidence that dynein is involved in α-synuclein degradation via autophagy. This study also suggests that the intracellular transport system may be a prime target for the development of new therapeutics for PD and other neurodegenerative disorders characterized by protein aggregation.
